# Targeting SOS1 overcomes imatinib resistance with BCR-ABL independence through uptake transporter SLC22A4 in CML

**DOI:** 10.1016/j.omto.2021.11.010

**Published:** 2021-11-20

**Authors:** Yanjun Liu, Chuting Li, Rui Su, Zhao Yin, Guiping Huang, Juhua Yang, Zhendong Li, Keda Zhang, Jia Fei

**Affiliations:** 1 Department of Biochemistry and Molecular Biology, Medical College of Jinan University, Guangzhou 510632, China; 2 Engineering Technology Research Center of Guangdong Province for Small Nucleic Acids Drug Development, Guangzhou 510632, China; 3 Antisense Biopharmaceutical Technology Co., Ltd., Guangzhou 510632, China; 4 Department of General Surgery, The First Affiliated Hospital of Jinan University, Guangzhou 510632, Guangdong, China; 5 College of Pharmacy, Shenzhen Technology University, Shenzhen 518118, Guangdong, China

**Keywords:** SOS1, imatinib sensitivity, resistance with BCR-ABL independence, CML, SLC22A4, RNA-seq

## Abstract

Resistance to the BCR-ABL inhibitor imatinib mesylate poses a major problem for the treatment of chronic myeloid leukemia. Imatinib resistance often results from a secondary mutation in BCR-ABL that interferes with drug binding. However, sometimes there is no mutation in BCR-ABL, and the basis of such BCR-ABL-independent imatinib mesylate resistance remains to be elucidated. SOS1, a guanine nucleotide exchange factor for Ras protein, affects drug sensitivity and resistance to imatinib. The depletion of SOS1 markedly inhibits cell growth either *in vitro* or *in vivo* and significantly increases the sensitivity of chronic myeloid leukemia cells to imatinib. Furthermore, LC-MS/MS and RNA-seq assays reveal that SOS1 negatively regulates the expression of SLC22A4, a member of the carnitine/organic cation transporter family, which mediates the active uptake of imatinib into chronic myeloid leukemia cells. HPLC assay confirms that intracellular accumulation of imatinib is accompanied by upregulation of SLC22A4 through SOS1 inhibition in both sensitive and resistant chronic myeloid leukemia cells. BAY-293, an inhibitor of SOS1/Ras, was found to depress proliferation and colony formation in chronic myeloid leukemia cells with resistance and BCR-ABL independence. Altogether these findings indicate that targeting SOS1 inhibition promotes imatinib sensitivity and overcomes resistance with BCR-ABL independence by SLC22A4-mediated uptake transport.

## Graphical Abstract

**Figure:**
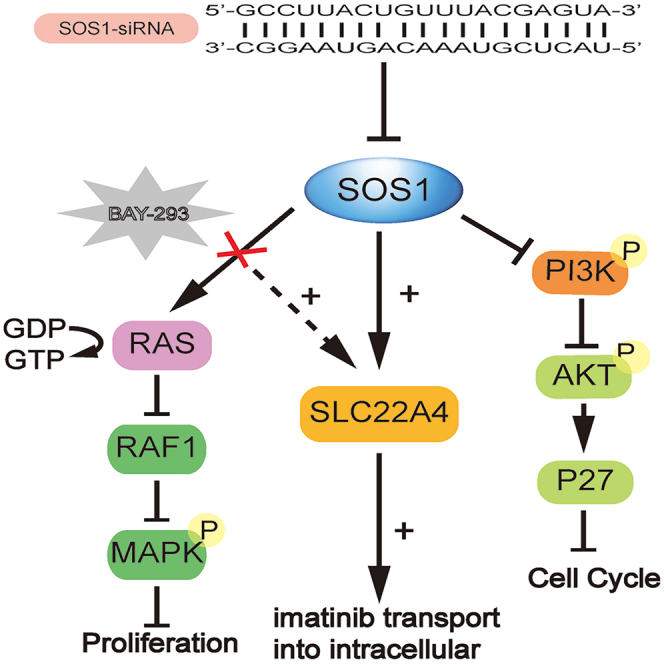

## Teaser

Liu et al. provide evidence that targeting inhibitor SOS1 promotes imatinib sensitivity and overcomes resistance with BCR-ABL independence by SLC22A4-mediated uptake transport.

## Introduction

Chronic myelogenous leukemia (CML) is characterized by a cytogenetic abnormality known as Philadelphia chromosome (Ph; BCR-ABL1), which causes malignant clonal hyperplasia of hematopoietic stem cells.^1^^,^^2^ Imatinib, a first-generation tyrosine kinase inhibitor (TKI), which targets the BCR-ABL fusion protein, has brought revolutionary change to therapy for chronic myelogenous leukemia.^3^^,^^4^ However, drug resistance appeared and became the main issue.^5^ Research has shown that resistance is due mostly to mutations in the BCR-ABL kinase domain.^6^ Among these, T315I mutation is frequent and the most resistant. With the deepening of research, drug resistance that is caused by mutations in BCR-ABL domains, including T315I, has been largely overcome.^7^^,^^8^ But some drug resistance is BCR-ABL independent, accounting for 50%,^9^ including CML stem cell-induced intrinsic resistance, which conveys a poor prognosis, and the basis of BCR-ABL-independent imatinib resistance is not understood.

A recent genetic study showed that the genetics of BCR-ABL-independent TKI resistance can largely vary among patients.^10^ Moreover, the genetic changes often suggest re-activation of multiple signaling pathways involved in CML pathogenesis.^10^^,^^11^ Son of Sevenless 1 (SOS1) is a guanine nucleotide exchange factor (GEF) for Ras protein.^12^ In CML cells, autophosphorylation of tyrosine 177 of BCR-ABL promotes the formation of a GRB2 complex with GAB2 and Son of Sevenless (SOS), followed by Ras activation.^13^^,^^14^ Sustained activation of the Ras/ERK signaling pathway caused malignant proliferation of cells.^15^^,^^16^ Recent studies have shown that SOS1 cooperates with STAT5 activation to initiate progenitor B cell leukemia and that SOS1 induces leukemogenesis in *KrasG12D* and contributes to BCR-ABL leukemogenesis.^17^, ^18^, ^19^ However, whether SOS1 is associated with drug response remains poorly understood.

In our previous research, SOS1 was a direct target gene of mir-181a, which was a prognostic marker in leukemia.^20^^,^^21^ Here, we found that targeting SOS1 increased the drug sensitivity of CML cells to imatinib by upregulating SLC22A4. SLC22A4, a protein that is permanently and thoroughly integrated into the cell membrane, is a member of the carnitine/organic cation transporter (OCTN) family.^22^ Drug disposition is affected by SLC22A4, which should be considered as a potential site of drug-drug interaction during the clinical development of new drugs.^23^, ^24^, ^25^ Gründemann et al.^26^ previously reported that SLC22A4 carried ergothioneine across the plasma membrane. Moreover, recent research showed that the active uptake of imatinib into cells was mediated mainly by the SLC22A4 transporter, and the different genotypes of the promoter of SLC22A4 were significantly associated with the imatinib response in CML.^27^, ^28^, ^29^ In this work, we attempt to provide a new therapeutic target for CML with BCR-ABL-independent imatinib resistance.

## Results

### Knockdown of SOS1 markedly inhibited the cell viability of CML cells *in vitro* and *in vivo*

**Figure 1:**
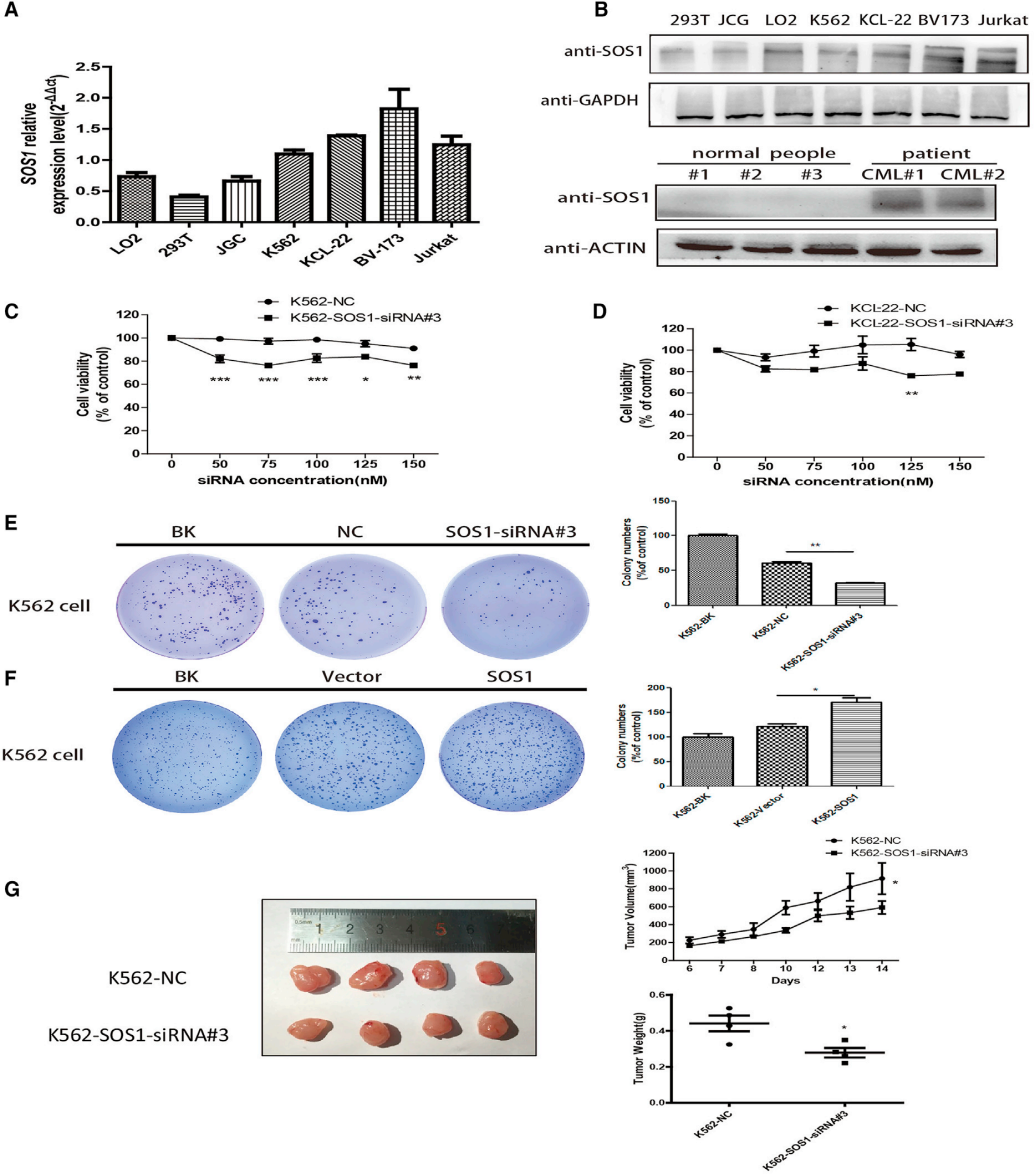
Knockdown of SOS1 markedly inhibited the cell viability of CML cells *in vitro* and *in vivo* (A) qPCR assays examined SOS1 mRNA levels in cells relative to GAPDH mRNA. (B) Top: expression screening was tested in protein level using western blotting. Bottom: in CML patients, expression of SOS1 can also be higher than in normal subjects. (C and D) Downregulation of SOS1 by transfection with SOS1-siRNA#3 significantly inhibited the cell viability of K562 cells, and the result was consistent with KCL-22 cells. (E) Downregulation of SOS1 by transfecting SOS1-siRNA markedly inhibited cell colony formation ability in K562 cells. (F) In contrast, SOS1 overexpression increased cell colony formation ability. (G) The tumor volume of the K562-SOS1-siRNA#3 group was significantly smaller than that of the K562-NC (negative control)-siRNA and K562-BK (blank) groups. Tumor weight in the two groups was recorded (n = 4 per group). Significance was determined using Student’s test comparing SOS1-siRNA#3 with NC-siRNA (p = 0.02 for volume, p = 0.03 for weight).

SOS1, a target of miR-181a, was confirmed by RNA immunoprecipitation (RIP) assays (Figure S1A). To confirm the oncogenic property of SOS1 in CML, protein and mRNA levels of SOS1 were detected in leukemia cells (K562, KCL-22, BV173, Jurkat, and peripheral blood mononuclear cells [PBMCs] of CML patients) and several normal human cells (293T, LO2, JCG, and PBMCs from normal subjects) by qPCR and western blotting (WB) assay. These results indicated that SOS1 was higher in leukemia cells than in normal cells (Figures 1A and 1B).

Next, we studied the functional consequences of SOS1 inhibition by SOS1-siRNA#3, which was chosen by western blot and qPCR from three candidate sequences (Figures S2A and S2B) in K562, KCL-22, and BV173 cells. The results showed that targeting SOS1 markedly restrained cell viability (Figures 1C and 1D) and colony formation ability (Figures 1E, S2D, and S2E) in K562 and KCL-22 cells. At the same time, SOS1 overexpression increased cell colony formation ability (Figure 1F). Additionally, silencing of SOS1 caused possible cell cycle arrest at G0/G1 in K562 and KCL-22 cells (Figures S3A and S3B). However, Figures S3A and S3B do not highlight this conclusion. The WB results in Figure S4D better indicate that knockdown of SOS1 depressed the activity of the PI3K-AKT signaling pathway, thereby increasing cell-cycle arrest in G0/G1. *In vivo*, subcutaneous tumor formation in BALB/c nude mice revealed that knockdown of SOS1 reduced tumor growth by comparing the size and the weight of tumors in the two groups, K562-NC (negative control) and K562-SOS1-siRNA#3 (Figure 1G), consistent with the *in vitro* result.

### SOS1 affected the drug sensitivity of CML cells to imatinib

**Figure 2:**
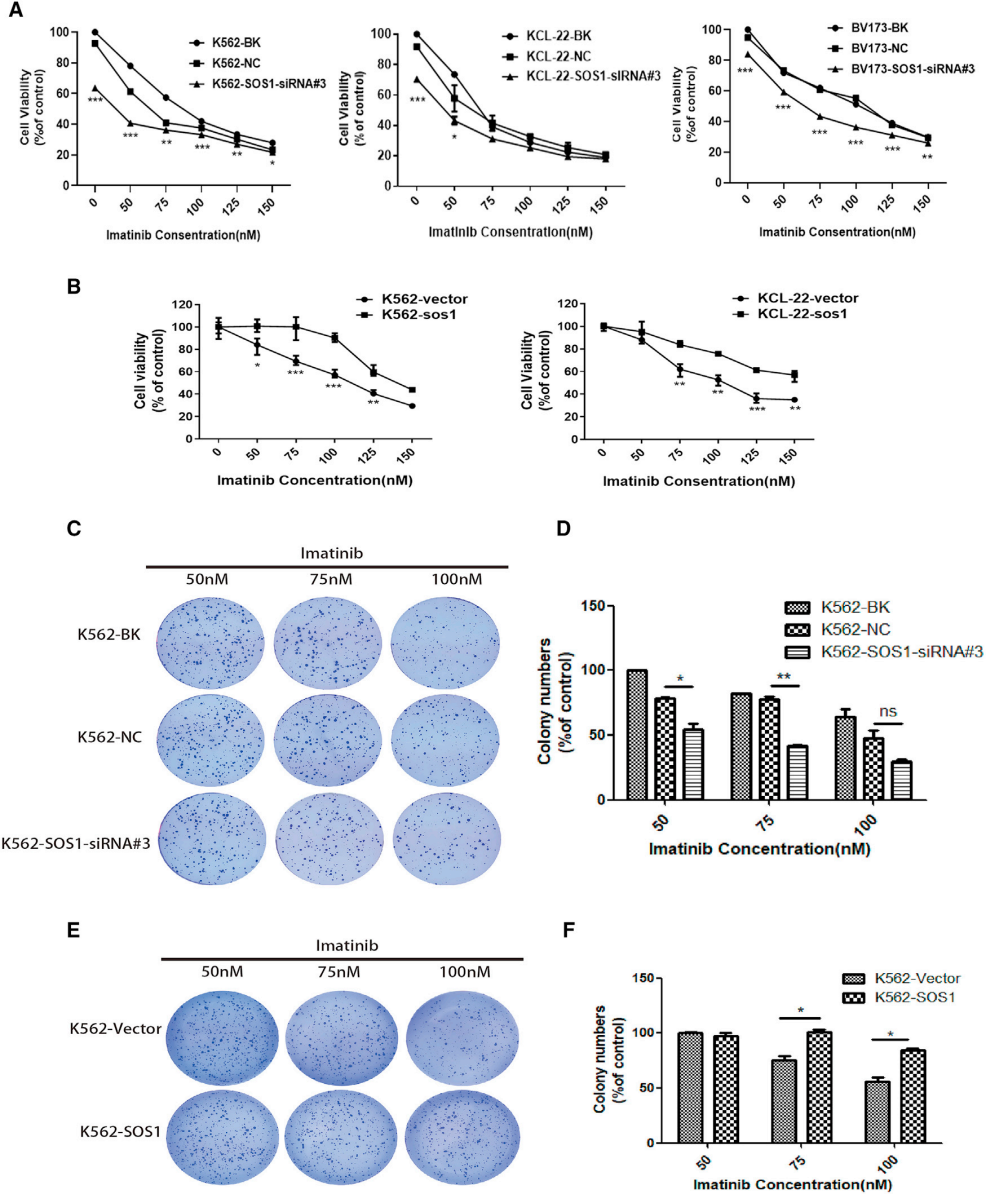
Silencing of SOS1 significantly increased the drug sensitivity of CML cells to imatinib (A) Downregulation of SOS1 by transfecting SOS1-siRNA#3 significantly increased the drug sensitivity of K562, KCL-22, and BV173 cells to imatinib. (B) On the contrary, SOS1 overexpression significantly enhanced cell resistance to imatinib compared with empty vector control. (C and D) Cells in which SOS1 was downregulated proliferated slowly under treatment with imatinib compared with the K562 and K562-NC groups. (E and F) However, cell colony formation ability was strengthened in SOS1-overexpressing cells. Data are presented as mean ± SD obtained from at least three independent experiments. Significance was determined using two-way ANOVA, ∗/∗∗p < 0.05 and ∗∗∗p < 0.01, SOS1-siRNA#3 versus NC, SOS1 overexpression versus empty vector control.

To explore the effect of SOS1 on imatinib sensitivity in CML, cell viability was detected in K562, KCL-22, and BV173 cells. As shown in Figure 2A, SOS1 knockdown significantly increased imatinib sensitivity of K562, KCL-22, and BV173 cells. On the contrary, SOS1 overexpression significantly enhanced cell resistance to imatinib compared with empty vector control (Figure 2B). However, overexpression of SOS1 by lentiviral infection to establish K562/KCL-22 cell lines stably expressing 3-FLAG-SOS1 (Figure S2C), decreased drug sensitivity. Soft agar assay also indicated that targeting SOS1 by SOS1-siRNA#3 reduced cell proliferation under treatment with imatinib (50, 75, 100 nM), compared with the K562-BK or K562-NC group (Figures 2C and 2D), and the result was consistent with KCL-22 cells (Figure S2F). In contrast, SOS1 overexpression promoted cell colony formation ability (Figures 2E and 2F). Thus, targeting SOS1 significantly increased the drug sensitivity of CML cells to imatinib.

### LC-MS/MS and RNA-seq assay revealed that knockdown of SOS1 promotes the expression of SLC22A4 in CML cells

**Figure 3:**
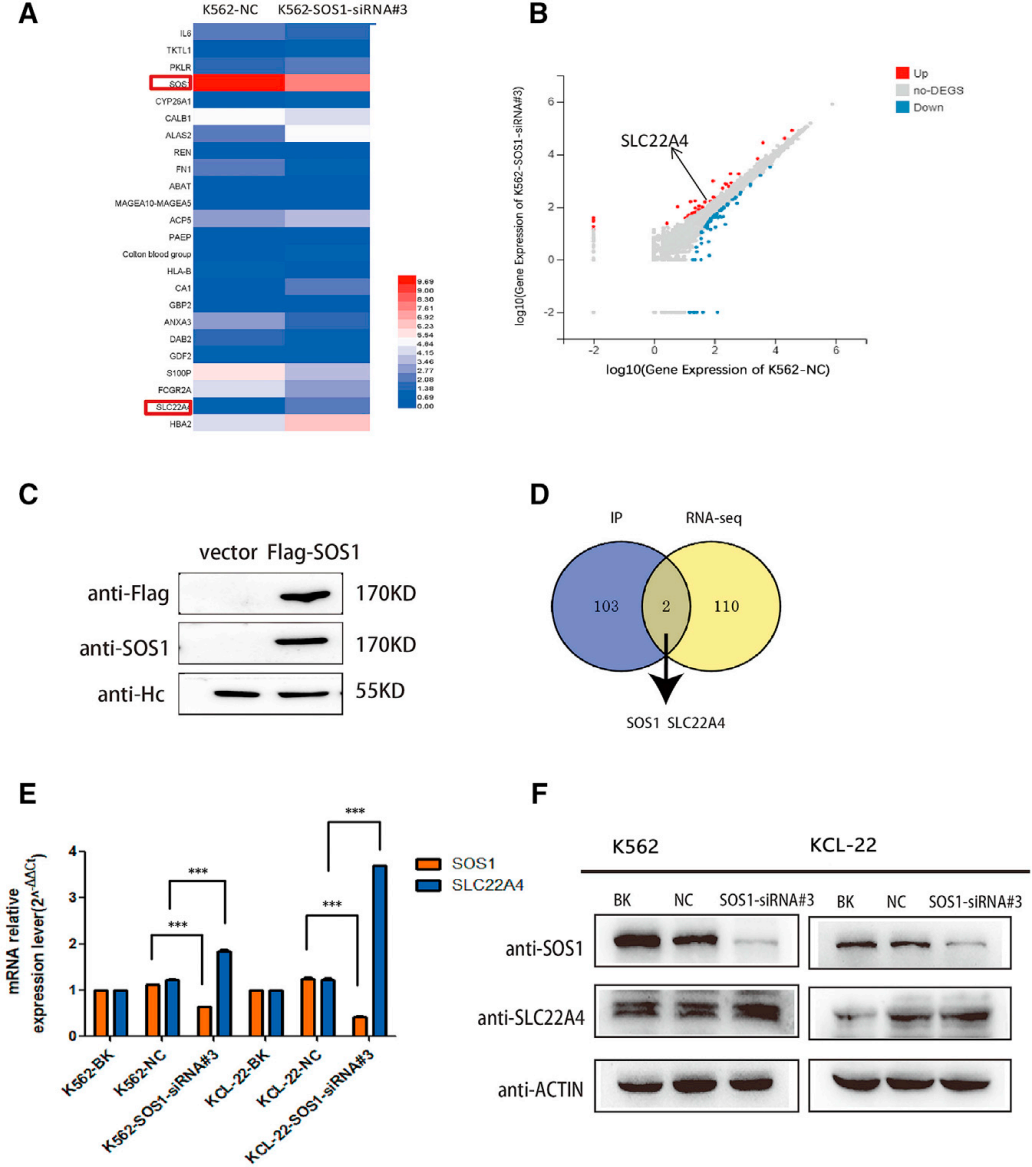
LC-MS/MS and RNA-seq assay revealed that knockdown of SOS1 promotes the expression of SLC22A4 in CML cells (A) A selection of differentially expressed genes are displayed in the Hop-map, in which SOS1 was downregulated and SLC22A4 was upregulated. (B) The Volcano plot also shows the upregulation of SLC22A4. (C) SOS1-interacted proteins were identified in purified precipitates and isolated by virtue of specific antibodies. (D) Venn diagram of LC-MS/MS after immunoprecipitation (IP) and RNA-seq shows that SOS1 interacted with SLC22A4. (E and F) The relationship of SOS1 and SLC22A4 was tested using qPCR and western blot in protein and mRNA levels, respectively.

To investigate the underlying molecular mechanism of SOS1, we constructed SOS1-overexpressed plasmid and transfected it to 293T cells, and the immunoprecipitates were separated by SDS-PAGE and analyzed using liquid chromatography-tandem mass spectrometry (LC-MS/MS). By comparing mass spectrometry results between the 293T flag-SOS1 group and the 293T-flag-empty groups, the protein interacting with SOS1 could be obtained. Proteins from mass spectrometry were integrated by KEGG (Kyoto Encyclopedia of Genes and Genomes) at the DAVID website (https://david.ncifcrf.gov/). KEGG pathway integration showed that SOS1 was related to the PI3K-AKT pathway (Figure S4A). Further research proved that the downregulation of SOS1 suppressed the PI3K-AKT pathway (Figure S4D). The results showed that a number of upregulated genes were identified, including SLC22A4 (Figures 3A and 3B). A number of SOS1-interacted proteins were identified in purified precipitates, isolated by virtue of specific antibodies (Figure 3C). Meanwhile, RNA sequencing (RNA-seq) assay was performed to correlate the relative expression of the gene in K562 after targeting SOS1 by siRNA transfection. After analyzing the results of LC-MS/MS and RNA-seq, SLC22A4 attracted our attention because it is one of the transporters of imatinib (Figure 3D). And this relationship of SOS1 and SLC22A4 was also confirmed using western blot and qPCR analysis (Figures 3E and 3F). Thus, we concluded that knockdown of SOS1 increased imatinib sensitivity by upregulating the expression of SLC22A4 in CML cells.

### HPLC assays confirmed that the increase of SLC22A4 promoted cellular imatinib uptake of CML cells

**Figure 4:**
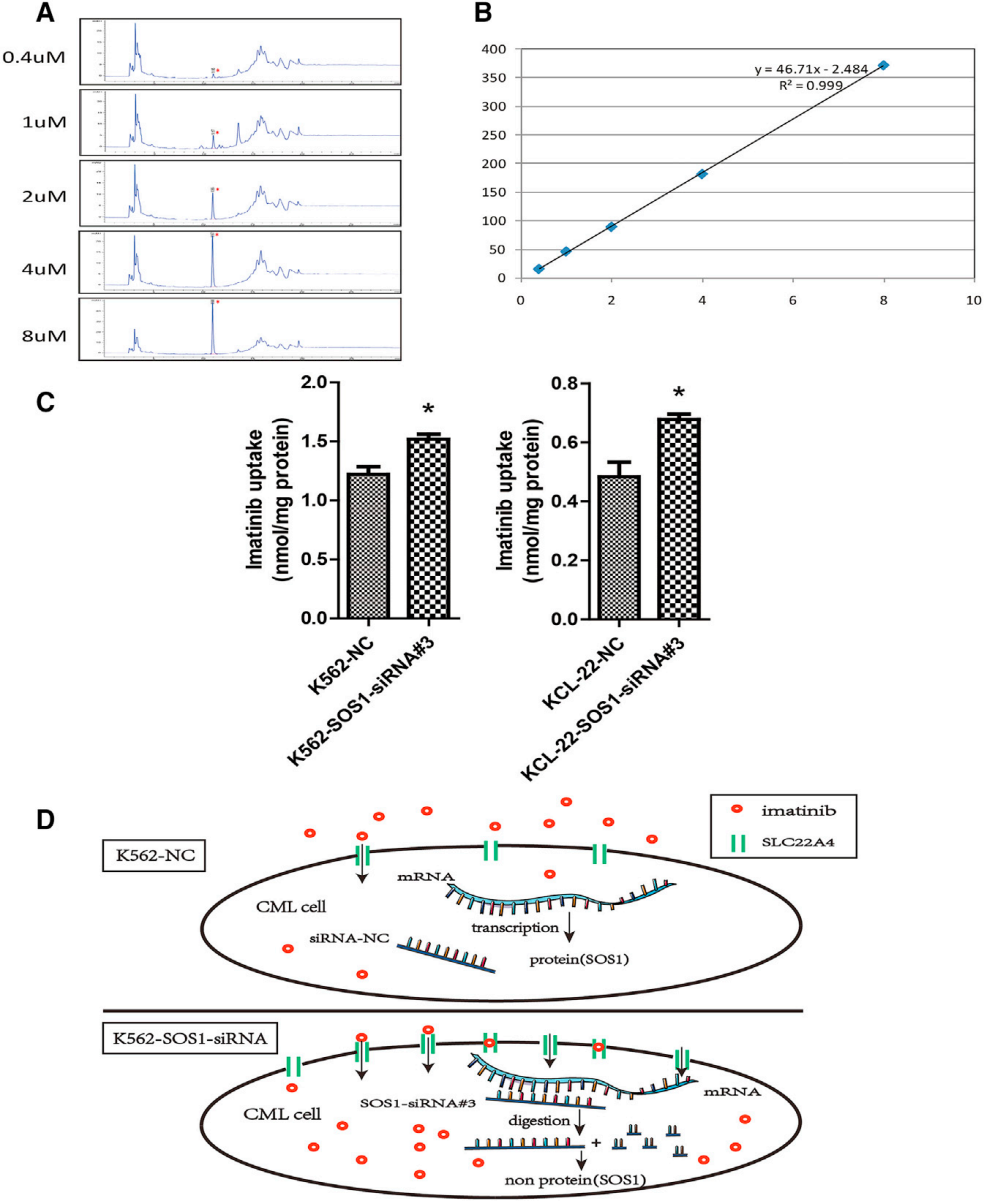
HPLC assays confirmed that the increase of SLC22A4 promoted cellular imatinib uptake of CML cells (A) Preparing different concentrations (0.4, 1, 2, 4, and 8 μM) of imatinib to draw a standard curve. High-performance liquid chromatography peak maps are shown above. (B) The standard curve was drafted according to the area of the peak map, and the R^2^ value of 0.999 approaches 1. (C) The relative content of imatinib in SOS1-knockdown cells increased in K562 and KCL-22 cells. (D) Diagram showing the relationship of SOS1 with SLC22A4. Data are presented as mean ± SD obtained from at least three independent experiments. Significance was determined using one-way ANOVA; ∗p < 0.05, SOS1-siRNA#3 versus NC.

On the basis of the findings above, we suggested that targeting SOS1 results in upregulating the expression of SLC22A4, thereby increasing the transport of imatinib, which ultimately leads to an increase in the imatinib sensitivity of CML cells. To validate this inference, we used high-performance liquid chromatography (HPLC) to detect the cellular content of imatinib in K562 cells in which SOS1 was targeted by siRNA. The results showed that targeting SOS1 promoted the uptake of imatinib by comparing the K562-SOS1-siRNA#3 and K562-NC groups (Figures 4A–4C). This indicated that targeting SOS1 increased imatinib sensitivity through upregulation of SLC22A4 in K562 cells. The mechanism is shown in Figure 4D.

### BAY-293 inhibited cell progression of cells with BCR-ABL-independent TKI resistance

**Figure 5:**
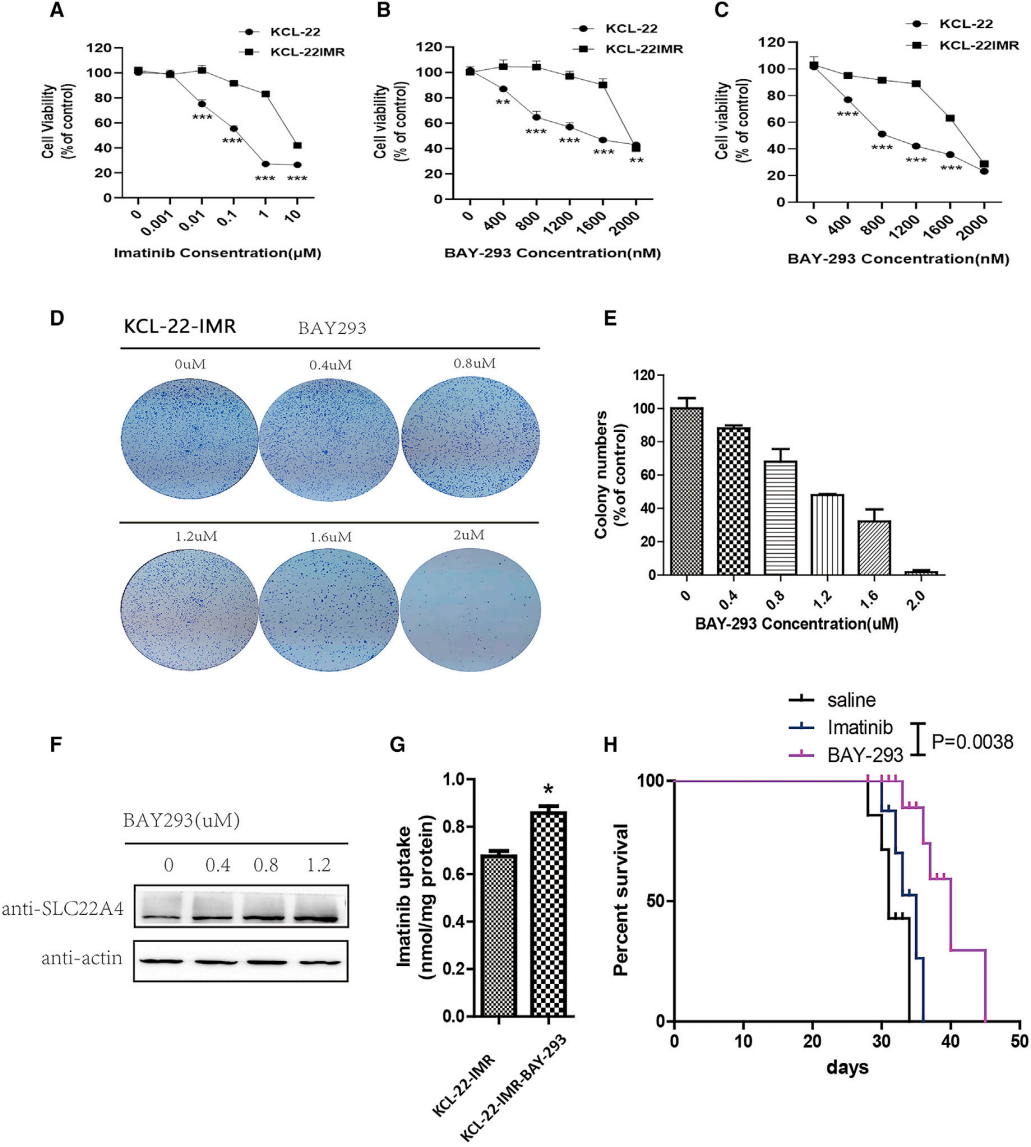
BAY-293 inhibited cell progression of BCR-ABL-independent TKI-resistant cells (A) KCL-22 and KCL-22-IMR cells were treated with different concentrations of imatinib. (B) BAY-293 in different concentrations (0.4, 0.8, 1.2, 1.6, and 2.0 μM) was cultivated in KCL-22 and KCL-22-IMR cells for 48 h. (C) KCL-22 cells were cultured at the IC_50_ of imatinib, and then BAY-293 at different concentrations was added into the cells for co-culture. The same was done for KCL-22-IMR cells. (D and E) The results showed that BAY-293 inhibited the colony formation ability of imatinib-resistant cells, and the higher the concentration of BAY-293, the stronger the inhibitory ability. (F) Western blot assay showed that BAY-293 inhibited SOS1 activity and promoted the expression of SLC22A4 simultaneously. (G) HPLC assay detected the intracellular content of imatinib in KCL-22-IMR and revealed that BAY-293 increased the absorption of imatinib. (H) BAY-293 prolonged CML survival in a mouse model of BCR-ABL-independent resistance compared with saline or imatinib (p = 0.0038). Data are presented as mean ± SD obtained from at least three independent experiments. Significance was determined using one-way ANOVA; ∗p < 0.05, KCL-22-IMR-BAY-293 vs KCL-22-IMR.

**Figure 6:**
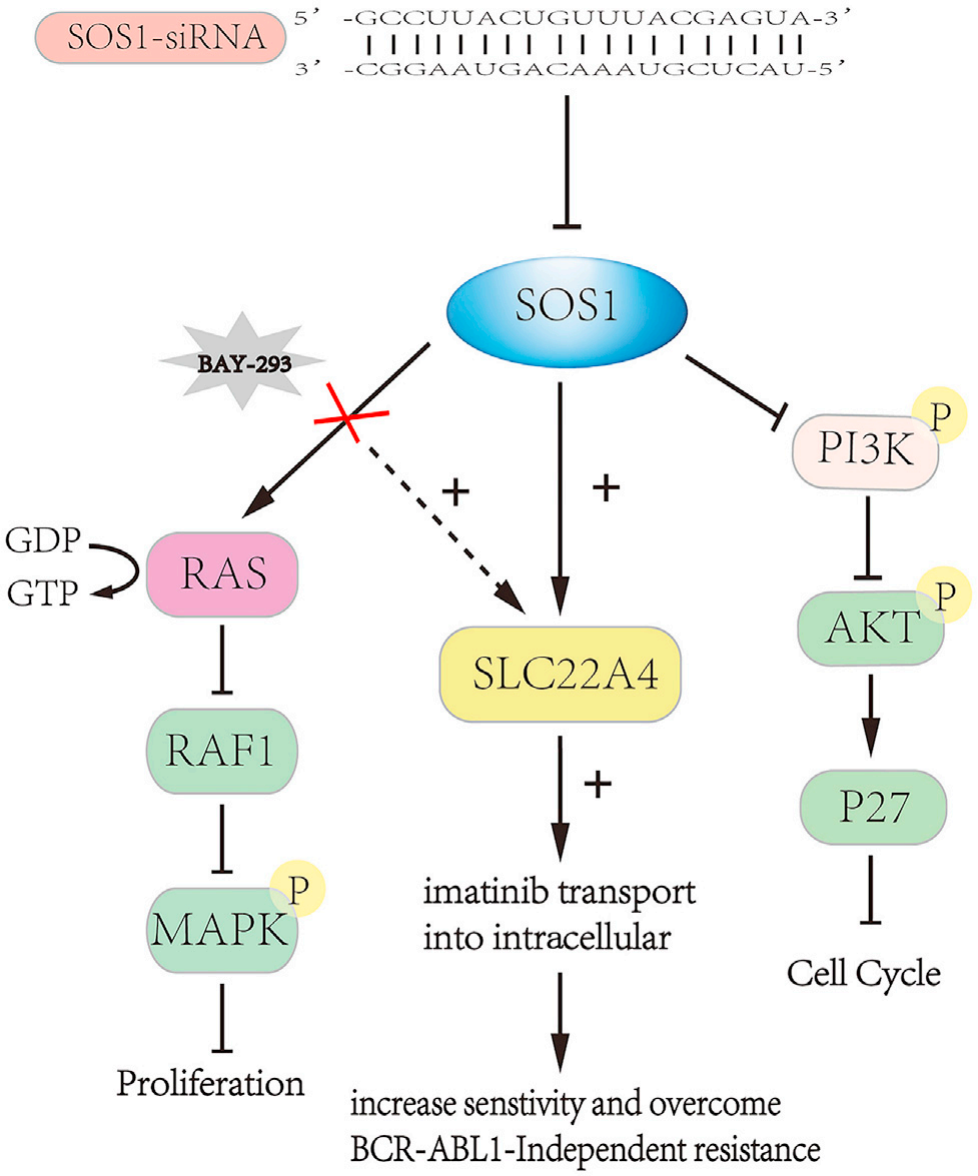
A summary diagram outlining the regulatory network of SOS1 in CML Targeting the inhibition SOS1 using siRNA arrested the cell cycle by depressing the PI3K-AKT signal pathway and increased drug sensitivity or overcame drug-resistance by promoting the expression of SLC22A4. BAY-293 is an inhibitor of SOS1, which blocks Ras activation and improves imatinib response through the expression of SLC22A4.

To further investigate the relationship of SOS1 and CML resistance, KCL-22 cells with BCR-ABL-independent imatinib resistance were developed by increasing the concentration of imatinib for a prolonged period. KCL-22 cells were more significantly inhibited by imatinib than KCL-22-IMR (imatinib-resistant) cells; the half maximal inhibitory concentration (IC_50_) of imatinib was 0.1976 μM in KCL-22 cells and 6.809 μM in KCL-22-IMR cells (Figure 5A). BAY-293, an inhibitor of SOS1/Ras that weakened the function of SOS1, inhibited cell proliferation both in KCL-22 and KCL-22-IMR cells in a dose-dependent manner (Figure 5B). At its IC_50_, imatinib combined with BAY-293 inhibited the proliferation of KCL-22 and KCL-22-IMR cells. Compared with KCL-22 and KCL-22-IMR cells, imatinib combined with BAY-293 suppressed KCL-22 cells more significantly (Figure 5C). Alone and in combination of imatinib, the IC_50_ of BAY-293 was 1.932 and 1.732 μM, respectively, in KCL-22-IMR cells. Therefore BAY-293 combined with imatinib showed little difference from BAY-293 alone. The IC_50_ of dasatinib is 0.2427 μM. Compared with BAY-293 or BAY-293 and imatinib in combination, the second-generation medicine in KCL-22-IMR cells (Figures S5A and S5B) was more effective than BAY-293 and BAY-293 and imatinib in combination. Dasatinib extended the lives of mice more effectively than the other treatment groups (Figure S5B). The colony formation ability of KCL-22-IMR cells was markedly decreased under treatment with BAY-293 (Figures 5D and 5E). We also found that BAY-293 promoted the expression of SLC22A4 simultaneously (Figure 5F). In order to confirm this phenomenon, we detected the intracellular content of imatinib using HPLC in KCL-22-IMR cells treated with BAY-293, and the results showed that BAY-293 increased the absorption of imatinib (Figure 5G). *In vivo*, BAY-293 prolonged CML survival in a mouse resistance model compared with imatinib (Figure 5H). This result suggested that BAY-293 overcome BCR-ABL-independent TKI resistance through upregulation of SLC22A4. A summary diagram outlining the regulatory network discussed above is presented in Figure 6.

## Discussion

There have been promising results in the treatment of BCR-ABL-dependent imatinib resistance, caused mainly by BCR-ABL mutation, which has been resolved following the discovery of second- and third-generation TKIs.^8^ However, in a proportion of imatinib-resistant CML patients, there is no mutation in BCR-ABL; this is classified as BCR-ABL-independent imatinib-resistance.^9^ BCR-ABL-independent TKI resistance is difficult to treat because of differences among individuals.^11^ Therefore, further research to identify therapeutic approaches capable of overcoming BCR-ABL-independent resistance is necessary.

SOS1, a guanine nucleotide exchange factor for Ras protein, plays an important role in the progress of BCR-ABL activation of the Ras/ERK pathway.^30^^,^^31^ However, a relationship between SOS1 and the drug sensitivity or resistance of imatinib was uncovered. In our previous research, SOS1 was a target of miR-181a-5p, a prognostic predictor in cancer, indicating that SOS1 was an oncogene in CML. Thus, we evaluated the expression of SOS1 in leukemia cells and patient samples at both the mRNA and protein levels, which was found to be higher than that in normal human samples.

The overexpression of SOS1 in CML led us to hypothesize that targeting SOS1 may show anti-CML activity and confer a therapeutic benefit in CML patients. K562 cells were transfected with SOS1-siRNA#3/NC-siRNA. After these transfected cells were transplanted subcutaneously into mice, repeated experiments showed that tumors in the K562-SOS1-siRNA#3 group were smaller than those in the K562-NC group because of the deletion of SOS1.We verified this hypothesis by targeting SOS1 using siRNA or BAY-293 and testing cell viability. Here we found that SOS1-siRNA/BAY-293 depressed imatinib-sensitive/resistant CML cell proliferation both *in vitro* and *in vivo*. At the same time, reducing the activity of SOS1 increased drug sensitivity and overcame BCR-ABL-independent TKI resistance.

The mechanism of SOS1’s anti-CML effect as identified using LC-MS/MS and RNA-seq is that the low regulation of SOS1 promotes expression of SLC22A4 in CML cells. On one hand, knockdown of SOS1 depressed the PI3K-AKT path, affecting the cell cycle. Chen et al.^32^ found that the proportion of SOS1 inhibition cells staying in G1 phase increased compared with the NC group in SOS1 inhibition, because activation of the PI3K-AKT signaling pathway promotes malignant cell proliferation induced by BCR-ABL. Our findings provide a rationale for therapeutic targeting of SOS1 levels to inhibit the proliferation of CML cells. Theard et al.^33^ demonstrated that the combined inhibition of EGFR and SOS1 significantly suppressed the Raf/MEK/ERK and PI3K/AKT signaling pathways. On the other hand, SOS1 interacted with SLC22A4, and the expression of SOS1 and SLC22A4 was negatively related. SLC22A4, a protein of the cell membrane, is a member of the carnitine/organic cation transporter family, which has been reported to mediate the active uptake of imatinib into cells.^29^ HPLC confirmed that the increase in SLC22A4 promoted the concentration of imatinib in CML cells. In this work, we suggest that inhibition of the activation of SOS1 increased the intracellular content of imatinib in both imatinib-sensitive and imatinib-resistant CML cells. This means that targeting SOS1 affects imatinib sensitivity and resistance with BCR-ABL-independence by upregulating SLC22A4 in CML cells. Angelini et al.^34^ reported that polymorphisms in OCTN1 and OCTN2 transporter genes are associated with prolonged time to progression in unresectable gastrointestinal stromal tumors treated with imatinib therapy, which was also confirmed in CML therapy. Other research revealed that OCTN helps overcome imatinib resistant CML.^35^ qPCR and WB assays showed that BAY-293 inhibited the expression of SOS1 and upregulated the expression of SLC22A4 to restrain the proliferation of KCL-22-IMR cells. In our study, we found that BAY-293, compared with imatinib, prolonged CML survival in mouse model of imatinib-resistant CML by reducing the activity of SOS1 and upregulating SLC22A4. In a mouse model of imatinib-resistant CML, the reason for the prolonged survival in the BAY-293 group was that the proliferation of KCL-22-IMR cells in the experimental group was inhibited by BAY-293 injection.

## Materials and methods

### Cells, patient samples, and imatinib-resistant cells

The K562 CML cell line was obtained from the Institute of Shanghai Cell Biology (China). KCL-22 and BV173 cells were kind gifts from Professor Markus Muschen (Children’s Hospital of Los Angeles, Los Angeles, CA). These cells were cultured in RPMI-1640 medium supplemented with 10% fetal bovine serum (FBS), 100 U/mL penicillin and 100 μg/mL streptomycin at 37°C in 5% CO_2_. Healthy or CML patient peripheral blood mononuclear cells were obtained from donors at Guangdong Provincial Emergency Hospital/Guangdong Second Provincial General Hospital after written informed consent was obtained according to institutional guidelines and the principles outlined in the Declaration of Helsinki. K562/KCL-22 cells were grown in increasing concentrations of imatinib for a prolonged period. We then developed an imatinib-resistant cell line with acquired BCR-ABL-independent resistance. Imatinib-resistant (K562-IMR/KCL-22-IMR) clones continued to proliferate when exposed to 1 μM imatinib.

### Plasmid and siRNA transfection

Lentiviral infection to establish K562/KCL-22 cell lines stably expressing 3-FLAG-SOS1 was performed according to the manufacturer’s instructions (FulenGen, Guangzhou, China). The sequences used in this study were as follows: negative control siRNA (NC-siRNA) and SOS1-siRNA (siRNA#1 sense: 5′-GCAGAATCTTCAC-CATCTA-3′; siRNA#1 antisense: 5′-UAGAUGGUGAAGAUUCUGC-3′; siRNA#2 sense: 5′-GTAGCAGTCTTAGAATACA-3′; siRNA#2 antisense: 5′-UGUAUUCUAA-GACUGCUAC-3′; siRNA#3 sense: 5′-GCCTTACTGTTTACGAGTA-3′; siRNA#3 antisense: 5′-UACUCGUAAACAGUAAGGC-3′). These RNA duplexes were synthesized and purified by Guangzhou Ribobio Company (Guangzhou, China) and stored at −20°C. RNA duplexes (50, 75, 100, 125, and 150 nM) were transfected into CML cells using Lipofectamine 2000 according to the manufacturer’s instructions. Knockdown efficiencies were quantified at the protein level using western blot.

### Real-time quantitative RT-PCR

Total RNA from CML cells lines was extracted using TRIzol (Invitrogen). cDNA was prepared with total RNA, 5 × qRT SuperMix, and RNase-free Water (all from All-in-one cDNA Synthesis SuperMix, Bimake). mRNAs were detected using SYBR-Green real-time PCR assays. Knockdown was quantified relative to GAPDH using the ΔΔCt method.

### Cell viability assay

Cell viability was determined using Cell Counting Kit-8 (CCK-8) assays. Briefly, K562 cells were seeded at a density of 1 × 10^5^ cells/mL in 96-well plates (100 μL/well). The cells were transfected with siRNA (50, 75, 100, 125, and 150 nM). After 6 h, the cells were treated with imatinib (50, 75, 100, 125, and 150 nM). Seventy-two hours later, 20 μL CCK-8 stock solution was added to each well, and the plate was incubated for 4 h at 37°C. Cell viability was assessed by measuring absorbance at 450 nm using a Bio-Tek microtiter plate reader.

### Soft agar assay

Agar (1.2 g) was dissolved in 100 mL water and kept at 50°C, and 2 × RPMI-1640 containing 20% fetal bovine serum was kept at 42°C. Agar and 2 × RPMI-1640 complete medium were mixed in equal volumes to a final concentration of 0.6% agar and 10% FBS in RPMI-1640, and 2 mL was poured in a six-well plate and allowed to solidify. Eight thousand K562/KCL-22 cells were transfected with SOS1-siRNA#3/NC-siRNA for 6 h and mixed in a volume of 1 mL RPMI-1640 complete medium, which contains 0.3% agar, and then poured over the already solidified agar basis. After the solution was cooled to room temperature and the top layer was solidified, the plate was transferred to the incubator at 37°C in 5% CO_2_. After 2 weeks, each well was stained with 0.5 mL 0.005% crystal violet for 1 h. Colonies were photographed.

### Subcutaneous CML model

All animals were housed at the Institute of Laboratory Animal Science of Jinan University. All animal procedures were conducted in accordance with the Guidelines for the Care and Use of Laboratory Animals. Six- to 8-week-old female BALB/c nude mice (Beijing Vital River Laboratory Animal Technology) were used for leukemogenesis experiments and maintained in a temperature- and humidity-controlled environment. A total of 1 × 10^7^ K562 cells transfected with SOS1-siRNA#3 or NC-siRNA were injected into the flank of the BALB/c nude mice. Tumor size was determined every day using a vernier caliper and expressed as absolute volume.

### Immunoprecipitation and western blotting

Cells were lysed in buffer containing 25 mM Tris-HCl (pH 7.4), 150 mM NaCl, 1% NP-40, 1 mM EDTA, 5% glycerol, phosphatase inhibitors, and protease inhibitors. After incubation on ice for 15 min, the lysates were transferred into microcentrifuge tubes and centrifuged at 13,000 × *g* for 15 min, and the resulting supernatants were transferred to new tubes for protein concentration measurement and immunoprecipitation. The protein concentration of the lysates was measured using Bradford dye (Takara), and equal amounts of protein were used for immunoprecipitation. For immunoprecipitation, anti-FLAG (1:100) (F3165, Sigma) or anti-SOS1 (1:100) (sc-376843, Santa Cruz) was added to the lysates for incubation overnight at 4°C, with rabbit IgG (1:100) as control antibody. Then Dynabeads Protein A was added for incubation for another 1 h at 4°C. After washing five times with the lysis buffer, the immunocomplexes were resuspended in protein loading buffer and analyzed using western blot. For western blot, ERK (#4695, Cell Signaling Technology), p-ERK (#9101, Cell Signaling Technology), AKT (C67E7, Cell Signaling Technology), p-AKT (D9E, Cell Signaling Technology), p27 (D69C12, Cell Signaling Technology), CDK4 (D9G3E, Cell Signaling Technology), CDK6 (DCS83, Cell Signaling Technology), β-actin (HRP-66009, ProteinTech), and cyclin D1 (E3P5S, Cell Signaling Technology) were added to the diluent, incubated overnight at 4°C, and finally exposed.

### LC-MS/MS analysis

In-gel digestion LC-MS/MS analysis was performed by Guangzhou Fitgene Biotechnology. The gel was cut into 48 slices, from which proteins were digested and resulting peptides extracted and lyophilized before further analysis. Peptide powders were resuspended in solvent A (2% acetonitrile, 0.1% formic acid in water) and loaded onto a C18 reverse-phase column (100 μm in diameter, 15 cm long, 3 μm resin from Michrom Bioresources, Auburn, CA). Each peptide mixture was separated with a linear gradient of solvent B (5%–15%) for 15 min, followed by a gradient from 15% to 35% for 85 min, and finally sustained at 90% for 20 min. Eluted peptides were injected directly on an LTQ-Orbitrap XL (Thermo Fisher Scientific) through a nano-electrospray ion source (Proxeon Biosystems) at a voltage of 1.85 kV and a transfer capillary temperature of 200°C. Data were acquired using Xcalibur software (Thermo Electron) in data-dependent mode. An accumulation of 106 ions was required to trigger a full MS scan, with a maximum accumulation time of 500 ms and a resolution of 60,000 (m/z 400), ranging from 400 to 2,000 Da. The six most intensive ions per MS scan were selected and fragmented by collision induced dissociation (CID) on the LTQ to perform the MS/MS scan, with an accumulation of at least 5,000 ions and a maximum accumulation time of 100 ms. The normalized collision energy was 35%, activation Q was 0.25, activation time was 30 ms, and dynamic exclusion was enabled with a maximum retention period of 90 s and a relative mass window of 10 ppm. A lock mass (PCM, MW445.12) was introduced to improve the mass accuracy of survey scans.

### RNA-seq assay

Total RNA was isolated and used for RNA-seq analysis. cDNA library construction and sequencing were performed by the Beijing Genomics Institute using the BGISEQ-500 platform. High-quality reads were aligned to the human reference genome (GRCh38) using Bowtie2. The expression levels for each of the genes were normalized to fragments per kilobase of exon model per million mapped reads (FPKM) using RNA-seq by expectation maximization (RSEM). Differentially expressed genes with fold change ≥ 2 were determined using the NOISeq method. The NOISeq R package is a comprehensive resource for the analysis of RNA-seq data, which can be divided into three blocks: count data quality control, filtering of low-count features, and normalization and batch effect correction and differential expression analysis. In each block, the package provides visualization diagrams and processing functions to help count datasets for comprehensive diagnosis and analysis.

### High-performance liquid chromatography analysis

Briefly, 3 × 10^5^ cells/well were transfected with SOS1-siRNA#3 or NC-siRNA (150 nM). After 48 h, each well was treated with imatinib (5 μM) for 10 min at 37°C.^36^ These cells were washed thrice with PBS and lysed on ice in cell lysis buffer for 20 min. Finally, cells were centrifuged at 13,000 rpm for 10 min, and supernatant was collected for protein quantification. And then the supernatant was mixed with the mobile phase and incubated overnight at 4°C, for precipitating protein. This mixture was centrifuged at 13,000 rpm for 10 min at 4°C, and supernatant was collected for HPLC analysis. HPLC analysis was performed on a ZORBX Carbohydrate Analysis column (4.6 mm inside diameter [ID] × 250 mm length) with the mobile phase consisting of acetonitrile, water, and H_2_PO_4_ (3:7:0.1) at a flow rate of 0.6 mL/min.^37^ The relative content of imatinib in each group was computed by competing with their protein concentration.

### Transplantation experiments

Experiments were performed and mice with disease sacrificed according to the guidelines of the Jinan University Animal Research Committee. Human KCL-22-IMR cells were transplanted via tail vein injection into 8- to 10-week-old female NOD/SCID mice (four to six mice were assigned per drug arm per experiment). Before injection, mice were irradiated with 2.5 Gy X-rays. After 1 week, mice were treated once daily via intraperitoneal (i.p.) injection for 7 days with vehicle control, BAY-293 (20 mg/kg), imatinib (50 mg/kg), and dasatinib (50 mg/kg) alone or in combination, and mice survival was observed and recorded. Survival analysis was performed using Kaplan-Meier analysis, and statistical significance was determined using the log rank test.

### Cell cycle assay

Equal amounts of KCL-22 and BV173 cells (3 × 10^5^ cells/well) were transfected with SOS1-siRNA#3 or NC-siRNA (150 nM). Cells were harvested after 72 h and washed with PBS three times. Cold ethanol (70%, 500 μL) was added to the cells at 4°C overnight in order to fix cells. Finally, cells were centrifuged at 500 × *g* for 10 min, and cells were collected for cell cycle analysis. Propidium iodide (PI)/RNaseA (500 μL; Cell Cycle Detection Kit, key gentec) staining solution prepared in advance was added, avoiding light at room temperature for 30–60 min. The cells were kept on ice in PI staining solution prior to cell cycle analysis using a BD Accuri C6 flow cytometer.

### RNA binding protein immunoprecipitation assay

Equal amounts of K562 cells were transfected with miR-181a-5p mimics or NC (100 nM). After 72 h, cells were collected and washed with PBS, and total protein was then extracted. Equal amounts of protein were used for immunoprecipitation. For immunoprecipitation, anti-AGO2 (1:100) was added to the lysates for incubation overnight at 4°C. Then Dynabeads Protein A was added for incubation for another 1 h at 4°C. After washing five times with the lysis buffer, the immunocomplexes were resuspended in PBS that contained protein kinase K at 55°C for 30 min. The supernatant was then collected for RNA extraction using TRIzol. The targeted mRNA of miR-181a-5p was detected using real-time PCR.

### Statistical analysis

Data are expressed as mean ± SD of a minimum of three biological replicates. Statistical analysis was carried out using GraphPad Prism version 5 (Systat Software, San Jose, CA). Student’s two-tailed unpaired t test, one-way ANOVA, and two-way ANOVA were used to determine significance, and p values < 0.05 were considered to indicate statistical significance.

## Supplemental information

Document S1. Figures S1–S5

Document S2. Article plus supplemental information
