# Supplementary material for: Targeting SOS1 overcomes imatinib resistance with BCR-ABL independence through uptake transporter SLC22A4 in CML
Source: Mol Ther Oncolytics. 2021 Nov 20;23:560–70. doi: 10.1016/j.omto.2021.11.010 (PMC8654699; doi:10.1016/j.omto.2021.11.010)
Supplement: Document S1. Figures S1–S5 [file mmc1.pdf]

OMTO, Volume *23*

Supplemental information

Targeting SOS1 overcomes imatinib resistance with BCR-ABL independence through uptake transporter SLC22A4 in CML

Yanjun Liu, Chuting Li, Rui Su, Zhao Yin, Guiping Huang, Juhua Yang, Zhendong Li, Keda Zhang, and Jia Fei

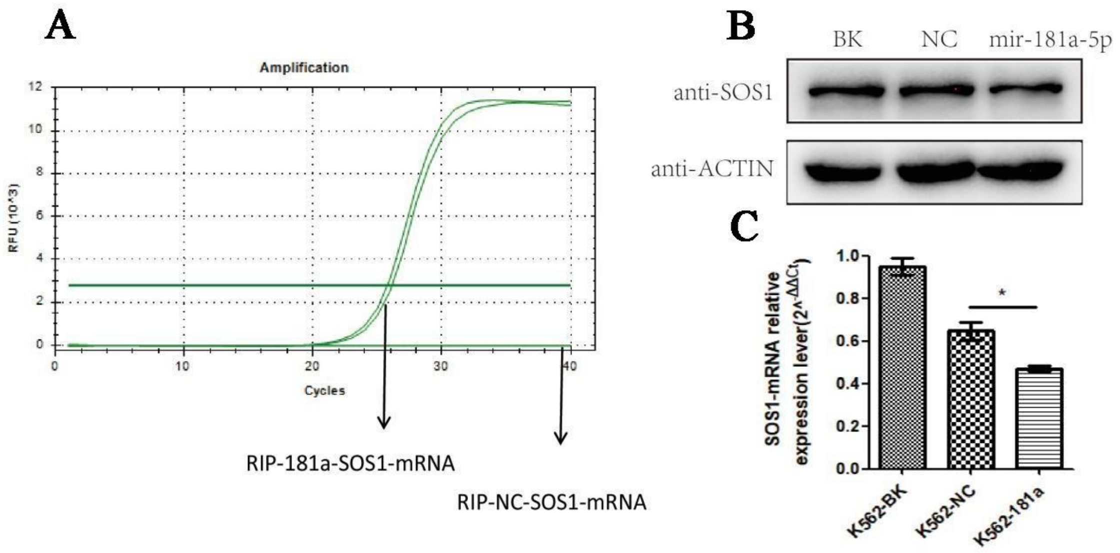

Supplement Figure 1. Confirming SOS1 was one target of miR-181a-5p. RIP conduct was tested the content of SOS1-mRNA through qPCR and the result show that SOS1-mRNA was test in K562-miR-181a-5p not in K562-NC (A). western blot (B) and qPCR (C) checked the relatively expression of SOS1 in K562 cell after transfected with miR-181a-5p mimic or NC, the result indicated that miR-181a-5p repressed the expression of SOS1. (The data were presented as the mean ± SD obtained from at least three independent experiments. Significance was determined by one-way ANOVA, *P<0.05, K562-miR-181-5p VS K562-NC.)

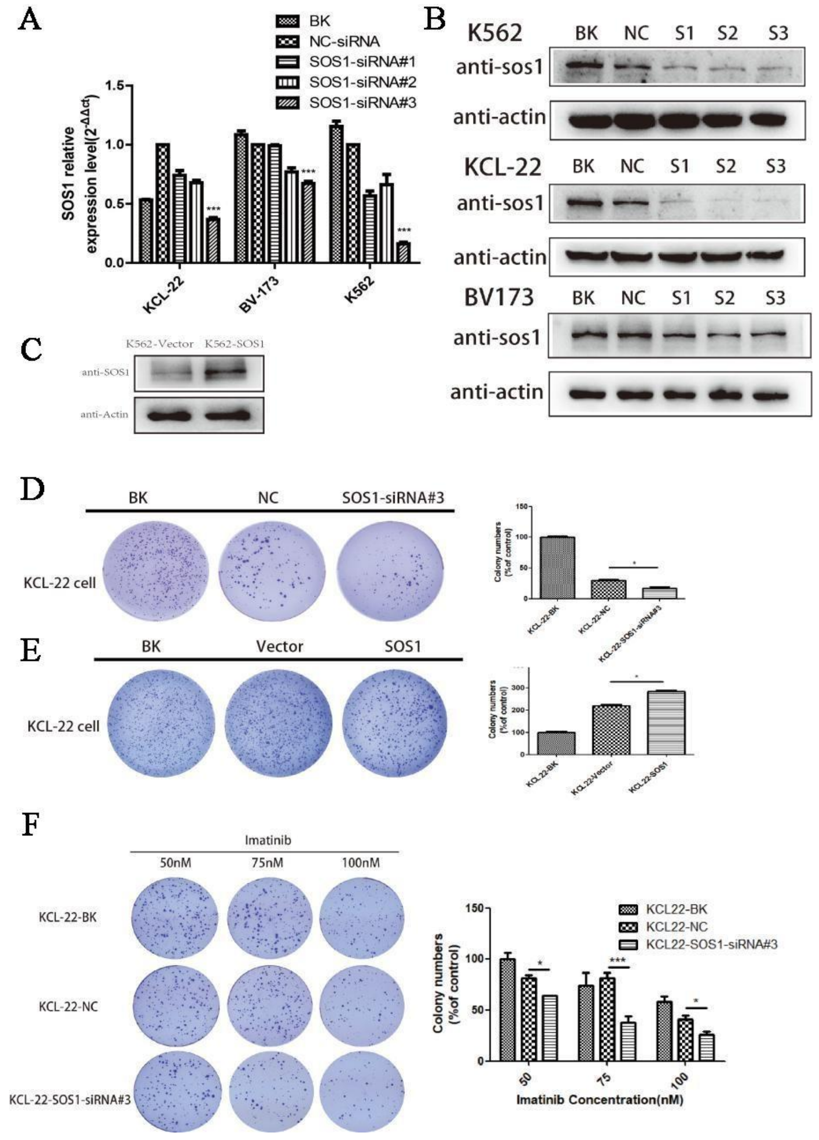

Supplement Figure 2. SOS1 siRNA valid sequence and its function. Screening of valid sequences of SOS1 siRNA from three siRNA which was synthetized randomly was analyzed by qPCR assays (A) and western blotting assays (B) in K562, KCL-22 and BV173 cells. Soft agar assay shown that SOS1-siRNA#3 restrained the colony-formation ability of KCL22 cell (D), and the overexpression of SOS1 in KCL22 cell, confirmed by western blot assay (C), enhanced this ability (E). Moreover, SOS1-siRNA#3 increased the drug sensitivity of KCL-22 cells to imatinib (F). (The data were presented as the mean ± SD obtained from at least three independent experiments. Significance was determined by one/two-way ANOVA, */**P<0.05, ***P<0.01 SOS1-siRNA#3 VS NC control, SOS1 overexpression VS empty-Vector control.)

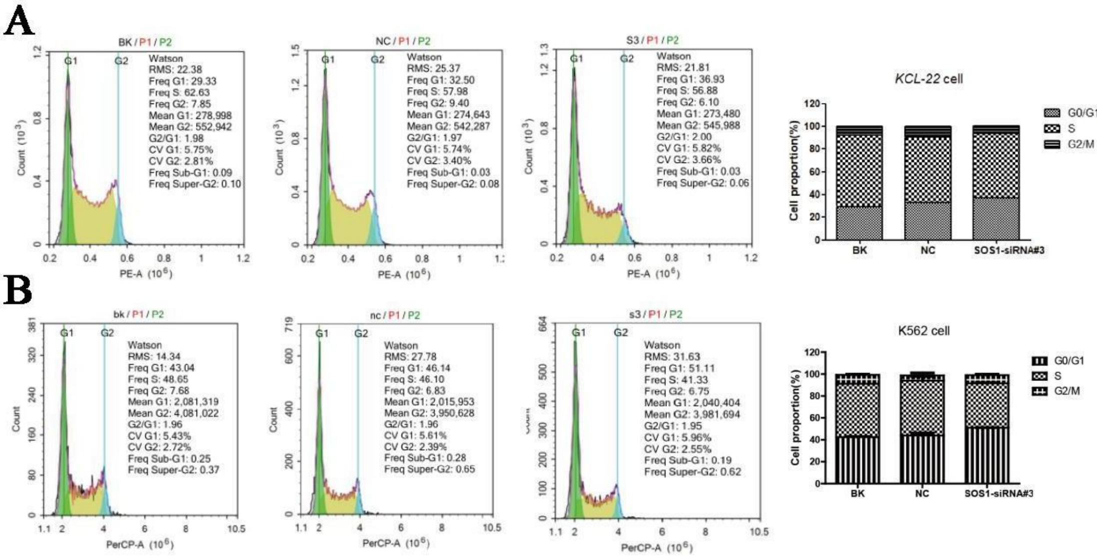

Supplement Figure 3. Knockdown SOS1 arrested cell cycle. (A and B) Equal amounts of KCL-22/K562 cells (3 × 10^5 cells/well) were transfected with SOS1-siRNA#3 or NC-siRNA (150 nM). Cells were harvested after 72h and washed with PBS for three times. Add 70% cold ethanol to the cells in 4℃ overnight in order to fix cells. Finally, cells were centrifuged at 500g for 10 min, and cells were collected for cell cycle analysis. Add 500 μL propidium iodide /RNaseA (Cell Cycle Detection Kit, keygentec) staining solution prepared in advance, avoiding light at room temperature for 30-60min. The cells were analysed by BD Accuri C6 flow cytometer. As is shown in the picture above, the cell cycle of the cells which tranfected with SOS1-siRNA#3 arrested at G0/G1 phase.

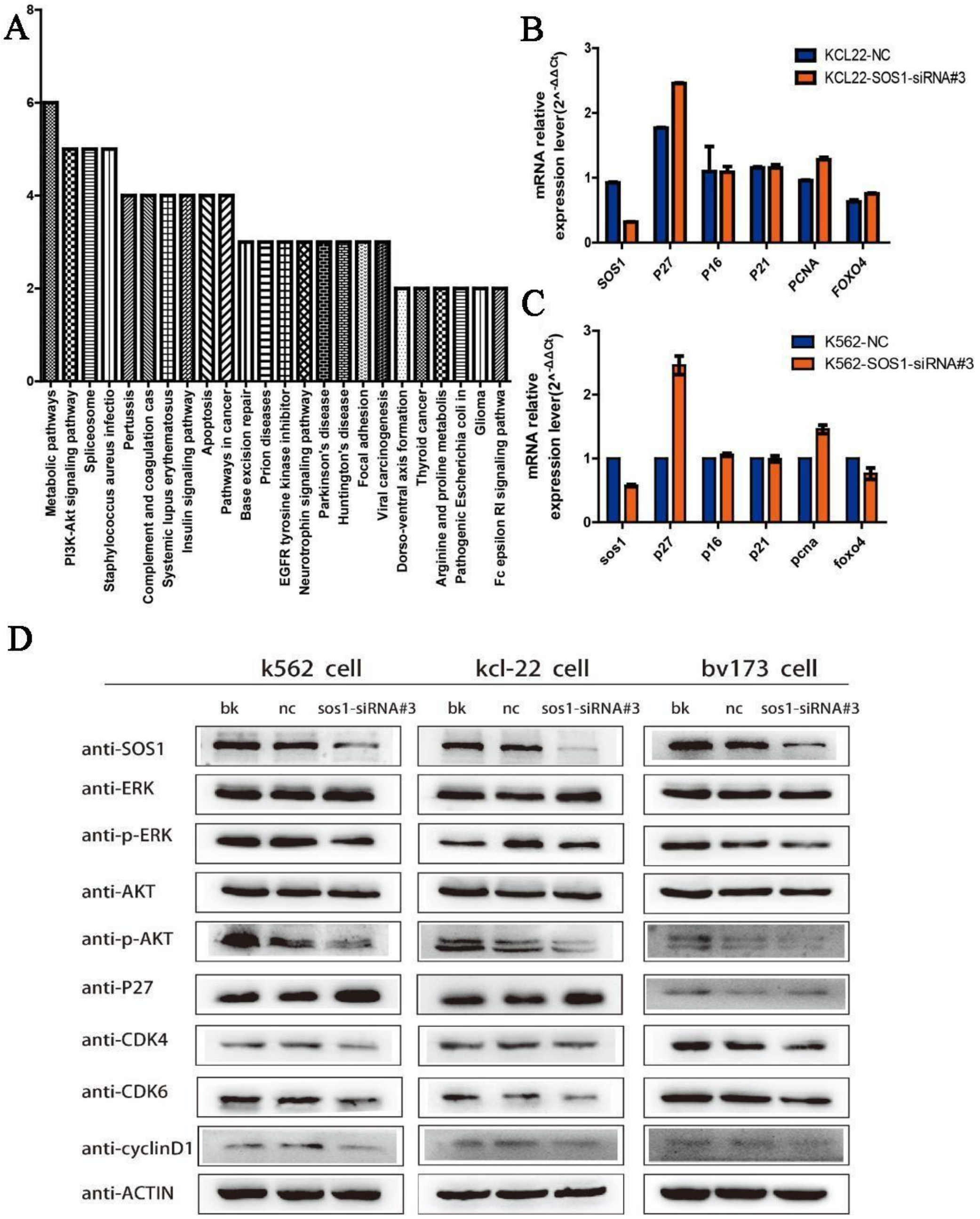

Supplement Figure 4. Down-regulation SOS1 depressed the PI3K-AKT pathway in CML cells. (A) KEGG pathway integration shown that SOS1 related to PI3K-AKT pathway which actives the cell cycle pathway, and we found that the down-regulation of SOS1 depressed the PI3K-AKT pathway. As we can see, the mRNA level of P27 was markedly increased both in KCL-22 and BV173 cells with the decrease of SOS1 (B and C). And we tested the change of the protein expression in the PI3K-AKT pathway, the result shown that the expression of p-ERK and p-AKT dropped but P27 increased and other cell cycle related protein were changed indicating cell cycle was arrested (D).

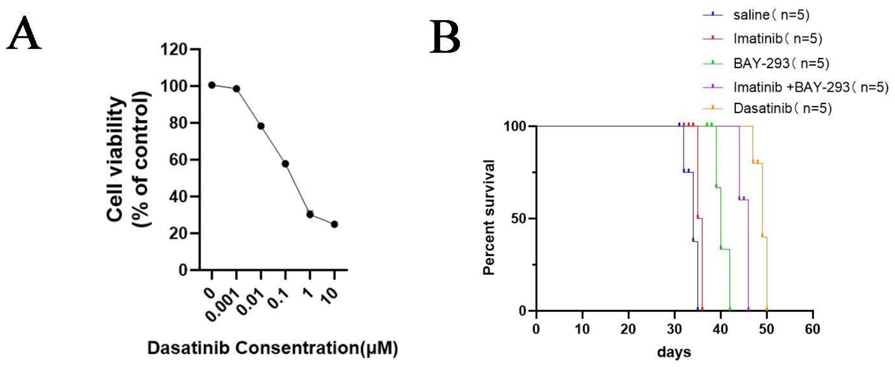

Supplement Figure 5. Dasatinib has certain inhibitory proliferation of imatinib resistant cells. (A) In a certain range, dasatinib inhibited KCL22-IMR cells proliferation more strongly with the increase of dasatinib concentration. (B)Survival curves were shown for mice injected with KCL22-IMR cells treated with imatinib, BAY-293, combination of imatinib and BAY-293, and dasatinib.
